# P-673. Post Hurricane Milton Surveillance for Respiratory and Gastrointestinal Illnesses

**DOI:** 10.1093/ofid/ofaf695.886

**Published:** 2026-01-11

**Authors:** Nychie Q Dotson, Julia Moody

**Affiliations:** HCA Healthcare West Florida Division, Tampa, FL; HCA Healthcare, Nashville, Tennessee

## Abstract

**Background:**

On October 9^th^, Hurricane Milton made landfall on Florida’s west coast as a category 3 hurricane bringing high winds, storm surge, catastrophic flooding, and extended power outages. As a result, communities navigated through water intrusion, subsequent boil water advisories, and operating on generator power. In response to Hurricane Milton’s impacts, active surveillance was implemented to monitor for increases in respiratory and gastrointestinal (GI) illnesses to assist with timely reporting and implementation of infection prevention (IP) practices.

**Table 1:**
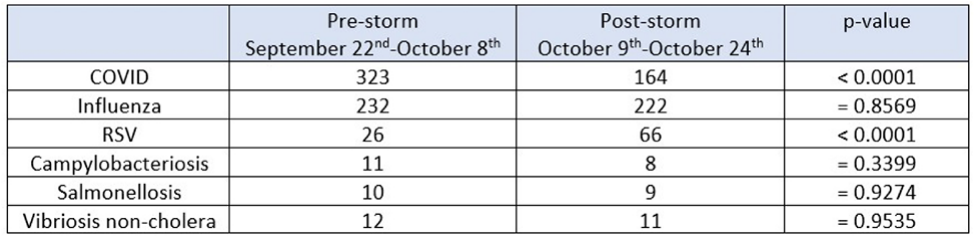
Identified respiratory and GI cases

**Table 2:**
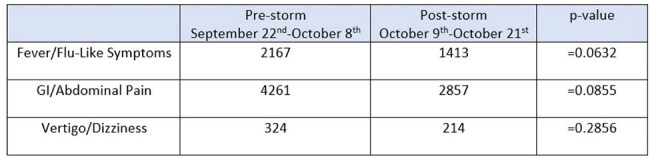
Chief complaints presenting to ER

**Methods:**

Immediately post-storm, active surveillance was initiated across 15 hospitals and 32 emergency room (ER) locations. Daily reports produced by corporate information technology teams provided remote electronic IP surveillance support. Utilizing an electronic surveillance application, respiratory viruses including COVID, influenza, respiratory syncytial virus (RSV), and GI illnesses including campylobacteriosis, salmonellosis, and vibriosis non-cholera were monitored. ER volumes were monitored assessing for chief complaints to include GI/abdominal pain, influenza-like symptoms, and vertigo.

**Results:**

During the immediate post-storm recovery phase, hospitals and ER locations saw a decrease in the number of COVID infections (p< 0.0001) while an increase in the number of RSV infections (p< 0.0001) was identified (Table 1). Monitored GI illnesses were not associated with an increase during the post-recovery phase. Decreases in ER presenting chief complaints of GI/abdominal pain, influenza-like symptoms, and vertigo were seen although not statistically significant (Table 2).

**Conclusion:**

Decreases in chief complaints of respiratory or GI illnesses during the post-storm phase may be explained by various factors including storm recovery activity and accessibility to seek medical care. Florida experiences a longer RSV season, with central Florida’s season occurring from August to March. The increase in RSV is consistent with increasing regional activity. As a part of the remote surveillance support, local infection prevention teams were able to reallocate time and support for hurricane remediation and recovery.

**Disclosures:**

All Authors: No reported disclosures

